# The Effectiveness of Pyruvic Acid Peeling in Improving the Quality of Life of Patients with Acne Vulgaris

**DOI:** 10.3390/jcm12103592

**Published:** 2023-05-22

**Authors:** Beata Jankowska, Małgorzata Elżbieta Zujko

**Affiliations:** 1 Department of Cosmetology, Faculty of Health Sciences, Łomża State University of Applied Sciences, Akademicka 14, 18-400 Łomża, Poland; bjankowska@ansl.edu.pl; 2 Department of Food Biotechnology, Faculty of Health Sciences, Medical University of Białystok, Szpitalna 37, 15-295 Białystok, Poland; 3 Department of Dietetics, Faculty of Health Sciences, Łomża State University of Applied Sciences, Akademicka 14, 18-400 Łomża, Poland

**Keywords:** acne vulgaris, quality of life, cosmetology procedures

## Abstract

Improving the quality of life (QoL) of patients with various chronic diseases has become a challenge and priority of contemporary medicine. The aim of this study was to assess the impact of pyruvic acid peeling on the QoL of patients with acne vulgaris. The study group consisted of 200 young patients (mean age 23.04 ± 4.71) with acne vulgaris of mainly mild or moderate severity. Basic data about the patient were collected using a self-reported questionnaire. The assessment of QoL was carried out using standardized questionnaires: CADI (Cardiff Acne Disability Index), DLQI (Dermatology Life Quality Index), SWLS (Satisfaction With Life Scale), and BDI (Beck Depression Inventory). The cosmetic intervention consisted of chemical peeling with 35% pyruvic acid for acne lesions on the body and included four series repeated at 7-day intervals. This study demonstrated that acne vulgaris impairs the quality of life of young people. There were no significant differences between the severity of acne and the lifestyles of the subjects. The applied cosmetic procedure significantly decreased the severity of the acne and improved the quality of life of the patients.

## 1. Introduction

Acne vulgaris is one of the most common skin conditions, affecting more than 9% of the world’s population, with the highest incidence in adolescents (80–85%) and often persisting into adulthood (30%). The prevalence of acne ranges from 27% to 96% in cross-sectional studies of large populations across countries and age groups [1]. Acne occurs mainly on the face, upper arms, trunk, and back. In a cross-sectional study conducted in Polish high schools, facial acne was found in 75% of adolescents aged 15–19 years [2].

Acne vulgaris is a chronic disease of the pilosebaceous unit that causes noninflammatory lesions (open and closed comedones), inflammatory lesions (papules, pustules, and nodules), and varying degrees of scarring. The following interrelated mechanisms can be considered in the pathogenesis of acne: overproduction of sebum, hyperkeratinization of the follicular infundibulum, development of inflammation, colonization of *Cutibacterium acnes* in sebaceous glands, and genetic factors [3]. Exacerbating factors in the course of the disease include hormonal disorders, stress, an unhealthy diet, excessive exposure of the skin to ultraviolet radiation, the use of inappropriate cosmetics, smoking, and the use of certain medications (e.g., anabolic steroids, corticosteroids, and antidepressants) [4].

The presence of acne, especially on exposed parts of the body, has negative psychosocial effects, which are expressed in the following forms: poor self-esteem, anxiety, anger, embarrassment, depression, altered social interactions, and decreased academic and work performance. The psychosocial impact of acne affects more women than men. In general, acne impairs many aspects of quality of life (QoL). In recent years, QoL has become an important concept in the medical field, necessary for understanding the consequences of the disease and for the effective treatment and rehabilitation of patients [5].

The pharmacological treatment of mild to moderate acne mainly includes topical retinoids, benzoyl peroxide, azelaic acid, salicylic acid, or combinations of topicals. Oral antibiotics and hormonal therapies are recommended in more severe cases of acne [6]. 

Acne requires long-term treatment, and many patients do not experience satisfactory therapeutic effects, which is often associated with non-adherence to medical recommendations. Therefore, selecting an appropriate treatment method is very important [7]. Effective acne treatment often requires cooperation between a dermatologist and a cosmetologist. Cosmetic procedures, depending on the severity of the acne, can be an individual therapy or can support pharmacological treatment. Among the cosmetic procedures for acne, the following can be distinguished: chemical peeling, microdermabrasion, cavitation peeling, sonophoresis, iontophoresis, photodynamic therapy, and laser therapy [8,9]. 

Chemical peeling is one of the oldest cosmetic treatments and, despite the availability of new technologies, is still widely used due to its low cost and excellent results. Chemical peeling is the controlled wounding of the epidermis and dermis for skin improvement. Common peels include alpha hydroxy acids (water-soluble) and beta hydroxy acids (lipid-soluble). The alpha hydroxy acids (AHAs) are extracted from fruit, sugar cane, and milk. Tang and Yang [10] reported that the beneficial effect of AHAs depends on their concentration and exposure time. Chilicka et al. [11] studied the effectiveness of azelaic and pyruvic acid peels in the treatment of female adult acne. The authors showed that both acids were comparable in reducing the severity of acne, desquamation, and the oiliness of the skin. However, pyruvic acid tended to reduce oiliness to a greater extent than azelaic acid. In another study [12], the authors observed a slightly greater improvement in QoL scores with pyruvic acid compared with an azelaic acid peeling treatment. 

Pyruvic acid is an α-keto acid with keratolytic, antimicrobial, and sebostatic properties, which stimulates the formation of collagen, glycoprotein, and elastic fibers. Pyruvic acid in concentrations of 40–70% with short-term applications (2–4 min) is currently used as an exfoliating agent in inflammatory acne, moderate acne scars, greasy skin, actinic keratosis, and warts [13]. Keisham et al. [14] demonstrated the efficiency of 40% pyruvic acid in reducing comedones, papules, and hyperpigmentation but not acne scars in Indian patients. Lee et al. [15] reported that pyruvic acid is safe at low concentrations (to 40%) but in higher concentrations can lead to the risk of focal irritation or hot spots.

Some studies indicate the role of selected factors on the QoL of patients with acne vulgaris [16]; however, the impact of cosmetic procedures [12], especially AHAs [17], on QoL is rarely studied. 

Therefore, the aim of this study was to assess whether the application of chemical peeling in the form of 35% pyruvic acid can improve the QoL of patients with acne vulgaris.

## 2. Materials and Methods

### 2.1. Ethical Approval

This study was conducted in accordance with the Helsinki Declaration and Good Clinical Practice and was approved by the Ethics Committee of the Łomża State University of Applied Sciences (approval number: 517301, date of approval: 8 May 2017). Informed consent was given by all the participants in the study.

### 2.2. Study Population and Data Collection

The study population consisted of 200 patients (169 women and 31 men) with acne vulgaris, who agreed to participate in the study and met the study inclusion criteria (17–40 years, tolerance to pyruvic acid used in cosmetic intervention, the presence of acne without complications, lack of dermatological treatment in the last year, and no pregnancy or lactation). The exclusion criteria were as follows: <17 and >40 years, allergies to components of the peeling formula, active herpes lesions, bacterial or fungal infection, facial dermatitis, autoimmune skin disorders, keloids or hypertrophic scars, dermatological treatment in the last year, pregnancy, and lactation.

Patients were identified for the study from people visiting a cosmetic studio in Choroszcz in the period from May 2017 to November 2018, based on the diagnosis of a dermatologist and cosmetologist. The severity of acne was classified as mild, moderate, severe, or very severe, according to the Global Acne Grading Scale (GAGS) [18]. Currently, no universal acne grading system can be recommended. However, dermatologists usually use an acne grading scale that takes into account the number and type of acne lesions, the severity of the disease, anatomical sites, and scars [19].

Data on the sociodemographic status (age, sex, level of education, commune size, marital status, and working status), anthropometric information (weight and height), health behaviors and lifestyle (night sleep duration, smoking, alcohol drinking, and adherence to the principles of proper nutrition), and information about acne were collected using self-reported questionnaires. Physical activity was assessed using a short version of the International Physical Activity Questionnaire (IPAQ) and was classified into the following categories: low, moderate, and high physical activity [20]. Body mass index (BMI) was calculated as body mass in kilograms divided by squared height in meters (kg/m^2^) and interpreted according to WHO recommendations [21].

Dietary food and nutrient intakes were assessed using 3-day 24 h dietary recalls (two randomly selected weekdays and one weekend day) and the computer program Diet 6.0. The quality of the diet was measured using the Healthy Diet Indicator (HDI) based on World Health Organization (WHO) recommendations [22]. The following cut-off values were adopted: saturated fatty acids < 10% TE (total energy without energy from alcohol), free sugars < 10% TE, polyunsaturated fatty acids 6–10% TE, protein 10–15% TE, cholesterol < 300 mg/day, fiber ≥ 25 g/day, and fruits and vegetables ≥ 400 g/day. The HDI score ranged from 0 (the least healthy diet) to 7 (the healthiest diet) and was classified as follows: high (6–7 points), moderate (4–5 points), and low (0–3 points).

The assessment of QoL in acne vulgaris was carried out using the Polish version of standardized questionnaires: CADI (Cardiff Acne Disability Index), DLQI (Dermatology Life Quality Index), SWLS (Satisfaction With Life Scale), and BDI (Beck Depression Inventory).

The CADI consists of 5 items, with each question answered on a 4-point scale, scored from 0 to 3, resulting in a score range of 0 to 15. A higher score represents greater QoL impairment (grade of impairment: 0, no impairment; 1–5, mild; 6–10, moderate; and 11–15, severe impairment). Questions about feelings, social life, skin exposure, and overall severity are based on the impact experienced over the previous month. If one item was not answered, we scored zero for the item and took the completion score as the sum of the scores for the four items [23,24].

The DLQI is a 10-item questionnaire scored from 0 to 3, resulting in a score range of 0 to 30 (grade of impairment: 0–1, no effect at all on the patient’s life; 2–5, small effect; 6–10, moderate effect; 11–20, very large effect; 21–30, extremely large effect on patient’s life). The DLQI is designed for use in patients aged 16 years and over and concerns patients’ perceptions of the impact of skin diseases on different aspects of their health-related quality of life over the last week. A higher score means greater QoL impairment [25].

The SWLS is a 5-item scale designed to measure global cognitive judgment of satisfaction with one’s life. Respondents, using a 7-point Likert scale (7—strongly agree; 6—agree; 5—slightly agree; 4—neither agree nor disagree; 3—slightly disagree; 2—disagree; 1—strongly disagree), indicate their agreement with each item. The obtained results ranging from 5 to 35 points were converted into the sten scale. Results in the range of 1–4 of the sten (5–17 points) were interpreted as low, 5–6 of the sten (18–23 points) as average, and 7–10 of the sten (24–35 points) as high satisfaction with life [26,27].

The BDI is a 4-point scale consisting of 21 questions, which aims to measure somatic, affective, cognitive, and motivational symptoms along with depression level and severity within the previous week. The total score ranges from 0 to 63, with higher scores indicating greater severity of depressive symptoms. A total score of 0–13 indicated no depression, 14–19 mild, 20–28 moderate, and 29–63 severe depression [28,29,30].

### 2.3. Cosmetic Intervention

The cosmetic intervention consisted of chemical peeling with 35% pyruvic acid (pH = 1.3) for acne lesions on the body and included 4 series repeated at 7-day intervals. Pyruvic acid was selected for the interventional studies because of its keratolytic and antimicrobial properties, its ability to stimulate collagen production and the formation of elastic fibers, and its rarely reported side effects, such as burning, redness, and scaling of the skin [31].

Two months after the cosmetic intervention, a control assessment of the QoL of the patients who underwent the procedure was performed. Unfortunately, only 158 of the 200 people from the initial cohort took part in the final stage of the study (79%). Among the 42 excluded participants after the follow-up, 22 did not complete all of the standardized questionnaires, and 20 people resigned from the study without giving any reason.

**Figure 1:**
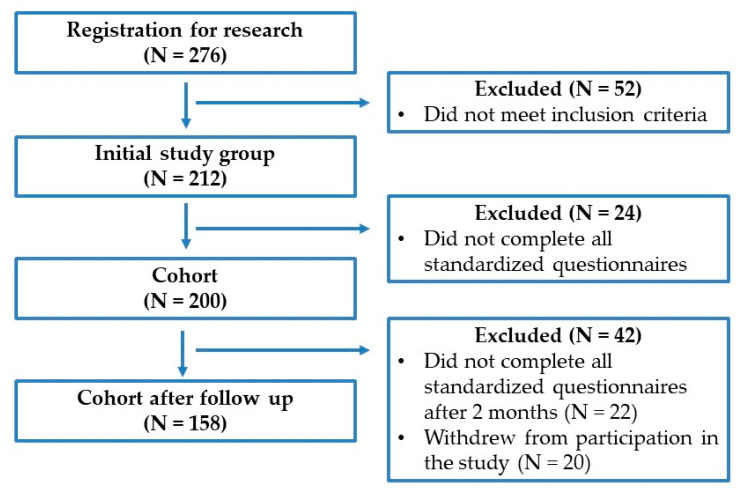
Flowchart of the study participants.

The flowchart of the study participants is presented in Figure 1.

The cosmetologist’s procedure during the intervention was as follows:

1. The procedure was performed with the patient lying down, on a bed secured with a disposable sheet, with a Lupa lamp turned on. During this time, the course of the procedure itself was explained, informing the patient about possible contraindications.
2. Next, a very thorough make-up removal was performed, removing the remains of sweat, sebum, and dirt from the skin to prepare it for peeling.
3. After the thorough cleansing of the skin, the skin was carefully examined under the Lupa lamp to determine where to apply the treatment.
4. The next stage of the procedure was to protect the corners of the mouth, eyes, and the most inflamed areas of the skin with petroleum jelly.
5. Before proceeding to the proper treatment, make-up was removed again, but with specialized preparations, intended for treatments with acids, changing the pH of the skin under the influence of acid. After cleansing, the acid was applied very vigorously to the affected areas for 30–60 s. The application lasted from 2 to a maximum of 8 min, depending on the severity of acne lesions and skin type.
6. The next step was to neutralize the acid and wash the preparation with cold water.
7. After the treatment, a serum, mask, and cream with a UV filter were applied. During this time, post-treatment recommendations were discussed with the patient, i.e., avoiding direct exposure to the sun, not using the solarium, not removing the exfoliating epidermis, and the systematic use of creams with a UV 50 filter (regardless of the weather), in order to prevent discoloration after the procedure. For home care, between successive treatments, it was recommended to use creams with a small amount of acid to better maintain the effects.
8. After the procedure, the patient was invited for another visit in 7 days.
9. The process of the intended exfoliation of the epidermis occurred approximately 2–3 days after the treatment. It was recommended to perform a series of pyruvic peeling treatments at approximately 1-week intervals. The break between successive treatments is the time necessary for the regeneration of the epidermis.

### 2.4. Statistical Analysis

Statistical analyses were performed using IBM SPSS Statistics v. 26 (SPSS INC., Chicago, IL, USA). Continuous variables are presented as the mean (M) and standard deviation (SD) and categorical variables as count (N) and percentage (%). The categorical variables were compared with Pearson’s chi-squared test. The normality of continuous data distribution was verified with the Shapiro–Wilk test and the Kolmogorov–Smirnov test with the Lilliefors correction. Comparisons of continuous variables between groups were conducted using the Mann–Whitney–Wilcoxon or Kruskal–Wallis tests. Correlations were calculated using the Spearman rank test. *p*-values of less than 0.05 were considered statistically significant. 

## 3. Results

The baseline characteristics of the study population are shown in Table 1. The mean age was 23.04 ± 4.71 years, 84.5% were women, 83% were single, 70.5% were learning, and 67% lived in a city. Most of the participants had a normal BMI (72.5%), middle level of education (68%), moderate level of physical activity (62%), and slept 7–9 h per night (55.5%). However, a large group of the participants were characterized by an unhealthy lifestyle: 18% were overweight, 36% had a low level of physical activity, and 37% slept less than 7 h. In addition, 30% were smokers and 52% drank alcohol once a week or more frequently. Among the respondents, 62% answered that they followed the principles of proper nutrition; however, their HDI was relatively low (HDI < 3). Moreover, the eating and food-buying habits of most people were incorrect.

The cosmetic characteristics of the study population are presented in Table 2. In the majority of respondents, the duration of acne was longer than 1 year (76.5%), with mild or moderate intensity (88.5%), and the location of acne lesions was on the face (97%). Among the types of acne lesions, comedones (88.5%), pustules (59%), papules (33%), and scars (23%) were the most common. Previous acne treatment at a dermatologist and a cosmetologist was declared by 54% and 48% of the respondents, respectively, and cavitation peeling was the most popular among cosmetology treatments. Only 30.5% of the respondents were satisfied with their previous treatment by the dermatologist or cosmetologist, and 69% removed acne lesions on their own. After the cosmetic procedure, 125 (62.5%) of the subjects were very satisfied with the effects of the treatment.

The association between the severity of acne and lifestyle is shown in Table 3. There were no significant differences between the severity of acne and the lifestyles of the subjects (BMI, level of physical activity, night sleep duration, smoking, alcohol drinking, and Healthy Diet Indicator score). 

The QoL measured by the CADI, DLQI, SWLS, and BDI before and after CP (cosmetic procedure) is shown in Table 4. It was found that the applied cosmetic procedure (chemical peeling with 35% pyruvic acid) had a significant (*p* < 0.001) effect in terms of lowering the impact of dermatological disease (DLQI) and acne (CADI) on the QoL and improving satisfaction with one’s own life (SWLS). No significant differences were found for BDI before and after CP. 

The correlations between SWLS and DLQI and CADI after CP are shown in Table 5. It was found that a lesser effect of dermatological disease and acne on a patient’s life is significantly associated with higher satisfaction with their life.

A comparison of acne severity before and after the cosmetic procedure is shown in Table 6. It was found that, after the CP, more participants had mild acne severity and fewer had moderate or severe acne severity compared to the stage before the CP.

## 4. Discussion

Acne vulgaris is a serious social problem in the population of young people, significantly affecting their quality of life. The impact of acne on the QoL is comparable with the impact of other noncommunicable diseases, such as diabetes and cardiovascular disease [32]. The psychosocial impact of acne is significantly related to the emotions, daily activities, social life, learning, and interpersonal relationships of adolescents [33]. Therefore, effective acne treatment should be associated with an improvement in the QoL of patients.

This study included 200 young participants, mainly women, mostly with mild to moderate acne severity (mainly comedones, pustules, and papules), a duration of acne of more than 1 year, and acne located on the face. A large group of respondents was characterized by an unhealthy lifestyle: every fifth person was overweight, every third person had a low level of physical activity, slept too-few hours at night, and smoked cigarettes, and half of the respondents drank alcohol once a week or more, and consumed sugar and sweets every day. Moreover, the quality of diet measured by the HDI score was relatively low. In addition, a large group of respondents had inappropriate eating and shopping behaviors. No significant differences were found between the severity of acne and lifestyle parameters. However, some authors have shown a relationship between an unhealthy lifestyle and the development of acne vulgaris [34]. Findings from the NutriNet-Santé Prospective Cohort Study (in which 24,452 participants completed an online self-questionnaire) show that the consumption of milk, sugary beverages, and fatty and sugary products was associated with current acne in adults [35]. On the contrary, other authors indicated no such relationship, but they emphasized the role of genes and hormonal determinants [36]. 

In a previous study, it was shown that patients with predominant comedonal and papulopustular acne should initially be treated with topical retinoids and acids (azelaic acid, salicylic acid, and pyruvic acid), with the treatment being tailored according to the severity of the disease [37]. Chemical peels are one of the most frequently used agents for acne treatment. Such treatment causes the controlled destruction of the epidermis and dermis, leading to the exfoliation and removal of superficial lesions, followed by the regeneration of new epidermal and dermal tissues [38]. In this intervention study, 35% pyruvic acid was chosen due to its high efficacy and rarely reported side effects [31].

The results of this research clearly show that the cosmetic treatment (chemical peeling with 35% pyruvic acid) significantly improved the QoL of patients, measured by the CADI, DLQI, and SWLS questionnaires. Other authors have also shown that selected cosmetic procedures have a significant impact on improving the QoL using DLQI and Skindex-29 questionnaires [11,33]. Chilicka et al. [17] showed that both azelaic acid and pyruvic acid have a positive impact on decreasing acne severity and improving QoL in young women undergoing treatment; however, pyruvic acid peeling was more effective with regard to the subjective assessment of physical symptoms affecting QoL.

It was found that a lower effect of dermatological disease (DLQI scale) and acne (CADI scale) on a patient’s life was significantly associated with higher satisfaction with their life.

Acne is associated with decreased appearance-related satisfaction, self-esteem, and self-confidence, and increased anxiety and depression. In our study, there was no significant improvement in the level of depression measured by the BDI scale after the cosmetic procedure. However, after the pyruvic acid peeling, mild depression decreased from 23.4% to 17.1%, and moderate depression from 6.3% to 4.4%. A meta-analysis of 42 studies found a significant association of acne with depression and anxiety [39]. The authors indicated that acne explained 4.60% and 6.25% of the variability in depression and anxiety, respectively. Depression and anxiety were more prevalent among people with acne than people without acne, and adults with acne than adolescents with acne. This suggests that adults experience more stress related to acne than adolescents.

It was found that the severity of acne significantly decreased after the cosmetic procedure. The effectiveness of cosmetic procedures, especially acid peeling, in the treatment of acne vulgaris has also been confirmed in other studies [40,41,42]. Acid peels have gained popularity in the treatment of acne vulgaris because they are relatively cheap and safe procedures. It is worth considering chemical peeling before treatment with oral pharmacological agents. However, the appropriate cosmetology procedure should be undertaken in cooperation with the dermatologist. In this study, 30% of participants were dissatisfied with previous cosmetic and dermatological therapy, but only 13.5% had previously used a chemical peel. After the current cosmetic procedure, 125 (62.5%) of the subjects were very satisfied with the effects of the treatment.

The present study also has some strengths and limitations. The strength of this study is that we used standardized questionnaires (CADI, DLQI, SWLS, and BDI) to assess the QoL of patients, and the study group was large (200 young people). In addition, there is little scientific research regarding the clinical value of cosmetic acids in improving QoL in people with acne. The main limitation of this study is that the study group in the final stage after follow-up represented only 79% of the initial group. It can be assumed that people dissatisfied with the effect of the cosmetic procedure resigned from the study. On the other hand, it should be emphasized that, in this study, only 9.5% of people reported dissatisfaction after the cosmetic procedure, and no one reported side effects. In summary, this direction of research is promising, and cosmetic treatments may be the first intervention before dermatological treatment. More research is needed to compare the effectiveness of different acids, at different concentrations, in improving the quality of life of patients with acne vulgaris.

## 5. Conclusions

In this study, acne vulgaris was found to impair the quality of life of young people. There were no significant differences between the severity of acne and the lifestyles of the subjects. The applied cosmetic procedure (chemical peeling with 35% pyruvic acid) significantly decreased the severity of acne and improved the quality of life of the patients. 

## Figures and Tables

**Table 1:** Baseline characteristics of the study population (N = 200).

Variables	Population
Age (year), M ± SD (range)	23.04 ± 4.71 (17–40)
Sex, N (%)	
Women	169 (84.5)
Men	31 (15.5)
BMI, N (%)	
<18.5 kg/m^2^	19 (9.5)
18.5–24.9 kg/m^2^	145 (72.5)
25.0–29.9 kg/m^2^	36 (18.0)
Level of education, N (%)	
Higher	53 (26.5)
Middle	136 (68.0)
Below middle	11 (5.5)
Commune size, N (%)	
City > 100,000 inhabitants	24 (12.0)
City 50,000–100,000 inhabitants	45 (22.5)
City < 50,000 inhabitants	65 (32.5)
Village	66 (33.0)
Working status, N (%)	
Working	55 (27.5)
Learning (school, university)	141 (70.5)
Not working and learning	4 (2.0)
Marital status, N (%)	
Married/cohabiting	34 (17.0)
Single	166 (83.0)
Level of physical activity, N (%)	
High	4 (2.0)
Moderate	124 (62.0)
Low	72 (36.0)
Night sleep duration, N (%)	
<7 h	74 (37.0)
7–9 h	111 (55.5)
>9 h	15 (7.5)
Currently smoking, N (%)	60 (30.0)
Alcohol drinking, N (%)	
5–7 times a week	22 (11.0)
2–4 times a week	40 (20.0)
Once a week	42 (21.0)
Occasionally	96 (48.0)
Subjective assessment of eating habits, N (%)	124 (62)
Healthy Diet Indicator, M ± SD	2.87 ± 1.34
Regularity of eating, M ± SD	43 (21.5)
Eating at night and between meals, M ± SD	112 (56.0)
Reading food labels, M (%)	102 (51.0)
Choosing foods according to their composition, M (%)	44 (22.0)
Choosing foods according to their nutritional value, M (%)	17 (8.5)
Daily consumption of sugar and sweets, M (%)	108 (54.0)

Data presented as mean ± standard deviation (M ± SD) or number and percentage (N, %), BMI—body mass index.

**Table 2:** Cosmetic characteristics of the study population (N = 200).

Variables	Population
Acne duration, N (%)	
<1 year	47 (23.5)
1–2 years	38 (19.0)
3–5 years	68 (34.0)
>5 years	47 (23.5)
Acne severity, N (%)	
Mild	78 (39.0)
Moderate	99 (49.5)
Severe	23 (11.5)
Location of acne lesions, N (%)	
Face	194 (97.0)
Neck	54 (27.0)
Back	68 (34.0)
Shoulders	16 (8.0)
Others	1 (0.5)
Types of acne lesions, N (%)	
Comedones	177 (88.5)
Papules	66 (33.0)
Pustules	118 (59.0)
Nodules	4 (2.0)
Scars	46 (23.0)
Previous treatment of acne by the dermatologist, N (%)	108 (54.0)
Previous treatment of acne by the cosmetologist, N (%)	96 (48.0)
Previous types of treatment by the cosmetologist, N (%)	
Cavitation peeling	71 (35.5)
Chemical peeling	27 (13.5)
Herbal peeling	13 (6.5)
Microdermabrasion	33 (16.5)
Iontophoresis	12 (6.0)
Satisfaction with previous treatments, N (%)	61 (30.5)
Self-removal of acne lesions, N (%)	138 (69.0)
Satisfaction with the current cosmetic procedure	
I am very satisfied	125 (62.5)
I expected more improvement	21 (10.5)
I am not satisfied	19 (9.5)
I do not know	35 (17.5)

Data are presented as number and percentage (N, %).

**Table 3:** The association between severity of acne and lifestyle (N = 200).

Lifestyle Parameters	Acne Severity			*p*-Value
	Mild	Moderate	Severe	
Overweight (BMI > 25), N (%)	14 (17.9)	18 (18.2)	4 (17.4)	0.445
Low physical activity, N (%)	28 (35.9)	36 (36.4)	8 (36.0)	0.388
Insufficient sleep time (<7 h), N (%)	29 (37.2)	37 (37.4)	8 (34.8)	0.325
Currently smoking, N (%)	23 (29.5)	30 (30.3)	7 (30.4)	0.478
Alcohol drinking (≥1 a week), N (%)	41 (52.6)	52 (52.5)	11 (47.8)	0.265
Healthy Diet Indicator, M ± SD	2.81 ± 1.46	2.93 ± 1.39	2.85 ± 1.52	0.542

Data are presented as mean and standard deviation (M ± SD) or number and percentage (N, %).

**Table 4:** Quality of life scores before and after cosmetic procedure (N = 158).

QoL Scores	Before CP	After CP	*p*-Value
CADI, M ± SD	8.51 ± 3.65	4.12 ± 2.94	<0.001
No impairment, N (%)	63 (39.9)	92 (58.2)	
Mild impairment, N (%)	44 (27.8)	29 (18.4)	
Moderate impairment, N (%)	46 (29.1)	33 (20.9)	
Severe impairment, N (%)	5 (3.2)	4 (2.5)	
DLQI, M ± SD	9.16 ± 2.93	5.12 ± 3.45	<0.001
No effect, N (%)	23 (14.6)	34 (21.5)	
Small effect, N (%)	43 (27.2)	64 (40.5)	
Moderate effect, N (%)	63 (39.8)	46 (29.1)	
Very large effect, N (%)	27 (17.1)	12 (7.6)	
Extremely large effect, N (%)	2 (1.3)	2 (1.3)	
SWLS, M ± SD	22.08 ± 6.03	25.61 ± 6.26	<0.001
Low satisfaction, N (%)	42 (26.6)	19 (12.1)	
Average satisfaction, N (%)	49 (31.1)	39 (24.7)	
High satisfaction, N (%)	67 (42.3)	100 (63.2)	
BDI, M ± SD	7.44 ± 6.75	6.03 ± 5.47	0.201
No depression, N (%)	108 (68.4)	121 (76.6)	
Mild depression, N (%)	37 (23.4)	27 (17.1)	
Moderate depression, N (%)	10 (6.3)	7 (4.4)	
Severe depression, N (%)	3 (1.9)	3 (1.9)	

Data are presented as mean and standard deviation (M ± SD) or number and percentage (N, %); CP—cosmetic procedure, CADI—Cardiff Acne Disability Index, DLQI—Dermatology Life Quality Index, SWLS—Satisfaction With Life Scale, BDI—Beck Depression Inventory.

**Table 5:** Correlations between SWLS and DLQI and CADI after cosmetic procedure (N = 158).

	SWLS	
	r	*p*-Value
CADI	–0.345	<0.001
DLQI	–0.312	<0.001

CP—cosmetic procedure, CADI—Cardiff Acne Disability Index, DLQI—Dermatology Life Quality Index, SWLS—Satisfaction With Life Scale.

**Table 6:** Comparison of acne severity before and after cosmetic procedure (N = 158).

Acne Severity	Before CP	After CP	*p*-Value
Mild	56 (35.4)	72 (45.6)	0.003
Moderate	89 (56.3)	75 (47.5)	
Severe	13 (8.3)	11 (6.9)	

CP—cosmetic procedure.

## Data Availability

All the data in this study are available upon request to the authors at the following e-mail addresses: malgorzata.zujko@umb.edu.pl or bjankowska@ansl.edu.pl.
